# Performing well but not appreciating it – A trait feature of anorexia nervosa

**DOI:** 10.1002/jcv2.12194

**Published:** 2023-09-20

**Authors:** Tine Schuppli Hjerresen, Mette Bentz, Ayna Baladi Nejad, Estelle Raffin, Kasper Winther Andersen, Oliver James Hulme, Hartwig Roman Siebner, Kerstin Jessica Plessen

**Affiliations:** ^1^ Child and Adolescent Mental Health Center Copenhagen University Hospital ‐ Mental Health Services CPH Copenhagen Denmark; ^2^ Danish Research Centre for Magnetic Resonance Centre for Functional and Diagnostic Imaging and Research Copenhagen University Hospital ‐ Amager and Hvidovre Copenhagen Denmark; ^3^ Department of Clinical Medicine Faculty of Health and Medical Sciences University of Copenhagen Copenhagen Denmark; ^4^ Medical & Science Novo Nordisk A/S Søborg Denmark; ^5^ Defitech Chair of Clinical Neuroengineering Neuro‐X Institute and Brain Mind Institute (BMI) Swiss Federal Institute of Technology (EPFL) Geneva Switzerland; ^6^ London Mathematical Laboratory London UK; ^7^ Department of Psychology University of Copenhagen Copenhagen Denmark; ^8^ Department of Neurology Copenhagen University Hospital Bispebjerg and Frederiksberg Copenhagen Denmark; ^9^ Division of Child and Adolescent Psychiatry Department of Psychiatry University Hospital Lausanne Lausanne Switzerland

**Keywords:** adolescence, anorexia nervosa, eating disorder, inhibition, perfectionism, reaction time

## Abstract

**Background:**

Despite advances in the etiology of anorexia nervosa (AN), a large subgroup of individuals does not profit optimally from treatment. Perfectionism has been found to be a risk factor predicting the onset, severity, and duration of AN episodes. To date, perfectionism has been studied predominantly by the use of self‐report questionnaires, a useful approach that may, however, be impacted by demand characteristics, or other distortions of introspective or metacognitive access.

**Methods:**

Here we circumvent these problems via a behavioral paradigm in which participants perform a modified Go/NoGo task, whilst self‐evaluating their performance. We compared a group of 33 adolescent females during their first episode of AN (age = 16.0) with 29 female controls (age = 16.2), and 23 adolescent girls recovered from AN (age = 18.3) with 23 female controls (age = 18.5). The controls were closely matched by intelligence quotient and age to the two clinical groups.

**Results:**

First‐episode AN and control participants performed equally well on the task (reaction time and errors of commission), whereas the recovered group displayed significantly faster reaction times but incurred the same error rate. Despite performing at least as good as and predominantly better than control groups, both clinical groups evaluated their performances more negatively than controls.

**Conclusion:**

We offer a novel behavioral method for measuring perfectionism independent of self‐report, and we provide tentative evidence that this behavioral manifestation of perfectionism is evident during first‐episode AN and persists even after recovery.

## Graphical abstract

Perfectionism has been found to be a risk factor predicting the onset, severity, and duration of anorexia nervosa episodes. To date, perfectionism has been measured predominantly using self‐report questionnaires, which is impacted by introspective or metacognitive abilities. We provide a measure that is independent of self‐report, and we show that a behavioral manifestation of perfectionism is evident during participants' first‐episode of anorexia nervosa and persists even after recovery.

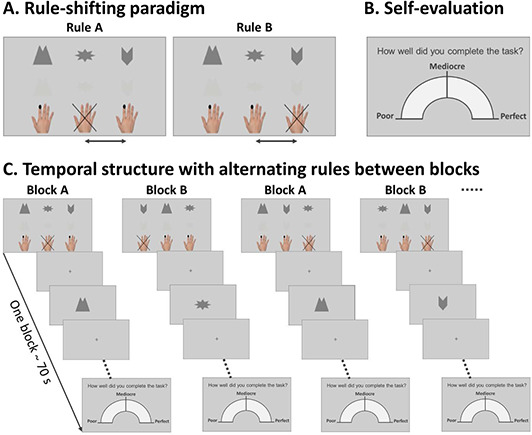

**Key points**

- Perfectionism has been found to be a risk factor predicting the onset, severity, and duration of AN episodes.
- This is the first study on behavioral perfectionism in AN combining a cognitive task with continuous self‐evaluation.
- The first‐episode AN group and the recovered group performed as good as or better than age‐matched controls, while evaluating their performance significantly more negatively than their respective controls.
- Correctly identifying subgroups of individuals suffering from AN with high levels of perfectionism may be an important step in individualizing treatment.

## INTRODUCTION

Anorexia nervosa (AN) is characterized by an excessive focus on food restriction, body shape and/or weight and these characteristics form the basis of the self‐evaluation for many individuals with AN (American Psychiatric Association, 2013; Blechert et al., 2011). When self‐evaluation builds upon a narrow and personally demanding foundation, it is associated with low self‐esteem, negative self‐concept, and clinical perfectionism (Fairburn et al., 2008; McFarlane et al., 2008; Shafran et al., 2002). These factors have been identified as risk factors for the onset of AN (Bulik et al., 2003; Farstad et al., 2016; Forsén Mantilla et al., 2014; Halmi et al., 2012; Holland et al., 2013; Kelly et al., 2014; Wade et al., 2008), to complicate treatment (Bizeul et al., 2001; Björck et al., 2007; Forsén Mantilla et al., 2019; Johnston et al., 2018; Petersson et al., 2021), and may affect the risk of relapse (Bardone‐Cone, Sturm, et al., 2010; Button & Warren, 2002; Nilsson et al., 2008; Srinivasagam et al., 1995). Hitherto, self‐evaluation has been primarily studied using questionnaires only with some exceptions (Mendoza et al., 2022). Questionnaires may suffer many of the difficulties associated with self‐report, such as demand characteristics, or relying on assumptions that participants can accurately introspect their own behaviors and feelings (Hofmann et al., 2005; Palmieri et al., 2021; Palminteri & Chevallier, 2018).

This perspective motivates the need to compliment psychiatric evaluations and self‐report with behavioral assays that may provide independent evaluation of perfectionistic traits. One of the few studies on behaviorally measured perfectionism reported that adults with AN spent more time on a text replication task and checked their answers more thoroughly on a bead sorting task than a control group (Lloyd et al., 2014). Findings in adults, however, may not translate directly to adolescents recently diagnosed with the disorder and it is not known whether recovered young individuals would display the same perfectionistic behavior. Furthermore, studies to date have not explored the participants' evaluation of own performance during the tasks in a meta‐perspective.

In line with the above, it has been proposed that individuals with AN will have longer reaction times (RTs) when performing tasks requiring cognitive control to minimize the number of errors (Bartholdy et al., 2016). Findings in AN populations, however, have not always reflected this statement (Bartholdy et al., 2017; Butler & Montgomery, 2005; Meule et al., 2011; Pieters et al., 2007; Rosval et al., 2006). The heterogeneity of the stimuli may partly explain the discrepant findings since emotionally salient cues tend to impact performance in participants with AN whereas neutral stimuli do not (Hildebrandt et al., 2015; Kullmann et al., 2014; Meule et al., 2011; Wierenga et al., 2014). In a recent study, adults recovered from AN displayed no differences compared to controls in their ability to inhibit their reactions (Oberndorfer et al., 2011), however evidence in recovered adolescents remains scarce.

We aimed to develop a novel method for behaviorally measuring perfectionism, and to test this in female adolescents with AN. We aimed to test this in first‐episode AN participants as well as participants recovered from the disorder to assess how persistent this behavioral perfectionism is across diagnostic state. If perfectionistic traits were present behaviorally in young females with a short duration of AN, and in young females recovered from AN, it would be most relevant to explore the possibilities of targeting perfectionism during treatment as this trait may affect treatment outcome and risk of relapse.

Our experimental strategy was to compare both performance metrics (RT and error rates) and self‐evaluation reports in the two AN groups with those of matched control groups, in a context unrelated to their psychopathology.

First, we hypothesized that both AN groups would have longer RTs and make fewer errors compared to matched controls. Second, we expected that the AN groups would evaluate their performance more negatively.

## METHODS AND MATERIALS

### Participants

We obtained study approval from the regional Scientific Ethical Committees (project number H‐2‐2012‐027) and The Danish Data Protection Agency and informed consent from participants and legal caretakers according to the guidelines of the Danish Health and Medicines Authority.

We included 33 females with “first‐episode” AN (AN^first^, ICD‐10: F50.0 or F50.1) (WHO, 1992). Participants in the AN^first^ group, younger than 18, were consecutively invited to participate as they presented for treatment at the Child and Adolescent Mental Health Services (CAMHS), Capital Region of Denmark, and participants, 18 years or older, were invited from Stolpegaard Psychiatric Center in a similar manner. AN^first^ participants were in their first episode of the disorder with a maximum duration of 1 year and had a low weight at study inclusion. We defined low weight as a body mass index (BMI) below the 25^th^ percentile corrected for gender and age for the 14‐ and 15‐year‐old participants and as a BMI below 18.5 for participants aged 16 and older. The BMI‐percentiles were based on a *z* score of each participant, considering the height, weight, age, and sex using the Center for Disease Control growth charts (Kuczmarski et al., 2002).

Twenty‐three participants were included in the “recovered” group (AN^rec^). They had been diagnosed with AN (ICD‐10: F50.0 or F50.1) in late childhood or adolescence and were invited on the basis of a previous CAMHS follow‐up study. AN^rec^ participants had a low weight at the beginning of treatment, were no longer in treatment for an ED, and had a good clinical outcome. We defined a good clinical outcome as a global score within one standard deviation of community norms on the Eating Disorder Examination (EDE) (Cooper & Fairburn, 1987; Fairburn, 2008), a score of nine or above on the Morgan‐Russell Outcome Assessment Schedule (MROAS) (Morgan & Hayward, 1988), and the absence of low weight for at least 1 year prior to entry into the study. The MROAS includes an overall score and subscales on ED symptoms, body weight, menstruation, other mental disorders, and age‐appropriate social functioning. We did not include the psycho‐sexual scale because it focuses on behaviors that were not relevant for a large group of the participants due to their young age.

Participants in the control groups were recruited through advertisements in the hospital's catchment area. The controls had no history of low weight, ED, or other mental disorder, and had no siblings with an ED. Transient childhood tics or adjustment disorders were not considered as criteria of exclusion. The controls were matched one‐to‐one on age to the participants in the clinical groups. Secondly, we matched the participants on intelligence quotient (IQ). IQ was measured with the Reynolds Intellectual Assessment Scales, Danish version (Reynolds & Kamphaus, 2011). Twenty‐nine controls (younger control group, CG^younger^) were matched to the AN^first^ group and 23 controls (older control group, CG^older^) to the AN^rec^ group, hence the two control groups consisted of different participants.

Comorbidities are common in AN. To ensure representativeness, we did not exclude based on past or present comorbidity of mental health disorders in the AN^first^ and AN^rec^ groups. The only exceptions were childhood autism (F84.0) and Asperger's syndrome (F84.5) since this study was part of a larger project examining social cognition (Bentz et al., 2017). Further exclusion criteria included preterm birth (before gestation week 37), head trauma with loss of consciousness, neurological illness, IQ below 70, not fluent in Danish, current use of psychotropic medication, and not being able to complete the test battery because of conditions such as an acute psychosis.

### Demographics

The AN^first^ participants and CG^younger^ were 16.0 (SD 1.6) and 16.2 (SD 1.7) years old, and the AN^rec^ participants and CG^older^ were 18.3 (SD 1.7) and 18.5 (SD 1.7) years old, respectively. The AN^first^ group displayed significantly lower BMI‐percentiles compared to the CG^younger^ (*t*(38.2) = −12.23, *p* < 0.001), whereas BMI‐percentiles did not differ across the AN^rec^ and CG^older^ groups (*t*(44) = −1.16, *p* = 0.252) (Table 1).

**TABLE 1:** Sample description.

	AN^first^	CG^younger^	AN^rec^	CG^older^	Test statistics	Pairwise comparisons
	Mean (SD)	Mean (SD)	Mean (SD)	Mean (SD)		
Age, years	16.0 (1.6)	16.2 (1.7)	18.3 (1.7)	18.5 (1.8)	ANOVA	AN^first^ versus CG^younger^, *p* = 0.690
					*p* < 0.001	AN^rec^ versus CG^older^, *p* = 0.688
Age at time of treatment	16.0 (1.7)	n/a	14.8 (1.5)	n/a	*t* test	
					*p* = 0.013	
Parents' highest education, years	15.8 (2.0)	15.7 (2.5)	14.5 (2.8)	15.1 (1.7)	ANOVA	AN^first^ versus CG^younger^, *p* = 0.800
					*p* = 0.119	AN^rec^ versus CG^older^, *p* = 0.360
Living with both parents together, *N* (%)	18 (54.6%)	19 (65.5%)	15 (65.2%)	10 (43.5%)	Chi‐square	AN^first^ versus CG^younger^, *p* = 0.380
					*p* = 0.349	AN^rec^ versus CG^older^, *p* = 0.139
BMI (kg/m^2^)	16.4 (1.2)	21.1 (1.8)	21.2 (1.8)	22.1 (2.5)	ANOVA	AN^first^ versus CG^younger^, *p* < 0.001
					*p* < 0.001	AN^rec^ versus CG^older^, *p* = 0.197
BMI *z* score corrected for age	−1.9 (1.0)	0.15 (0.5)	−0.1 (0.5)	0.1 (0.6)	ANOVA	AN^first^ versus CG^younger^, *p* < 0.001
					*p* < 0.001	AN^rec^ versus CG^older^, *p* = 0.266
BMI‐percentile^a^	8.0 (8.8)	55.3 (19.1)	47.1 (18.5)	54.2 (22.5)	ANOVA	AN^first^ versus CG^younger^, *p* < 0.001
					*p* < 0.001	AN^rec^ versus CG^older^, *p* = 0.250
EDE global score at time of treatment^b^	2.9 (1.6)	n/a	3.0 (1.2)	n/a	*t* test	
					*p* = 0.849	
EDI eating disorder risk composite, T‐score^c^	47.7 (11.1)	35.5 (6.5)	37.3 (6.3)	37.3 (7.0)	ANOVA	AN^first^ versus CG^younger^, *p* < 0.001
					*p* < 0.001	AN^rec^ versus CG^older^, *p* = 0.965
EDI perfectionism, T‐score^c^	43.3 (8.5)	42.1 (9.8)	41.8 (8.0)	39.5 (7.0)	ANOVA	AN^first^ versus CG^younger^, *p* = 0.626
					*p* = 0.437	AN^rec^ versus CG^older^, *p* = 0.296
Beck depression inventory for youth, T‐score	61.0 (11.1)	48.3 (9.1)	50.6 (12.1)	48.6 (8.0)	ANOVA	AN^first^ versus CG^younger^, *p* < 0.001
					*p* < 0.001	AN^rec^ versus CG^older^, *p* = 0.510
Beck anxiety inventory for youth, T‐score	57.5 (9.5)	48.4 (11.7)	51.7 (13.2)	49.7 (8.4)	ANOVA	AN^first^ versus CG^younger^, *p* = 0.001
					*p* = 0.006	AN^rec^ versus CG^older^, *p* = 0.530
General intelligence quotient, RIAS	107.5 (10.4)	111.5 (8.3)	101.9 (11.3)	104.9 (8.0)	ANOVA	AN^first^ versus CG^younger^, *p* = 0.100
					*p* = 0.004	AN^rec^ versus CG^older^, *p* = 0.303
EDE global score at time of study	2.9 (1.6)	n/a	0.7 (0.5)	n/a	*t* test	
					*p* < 0.001	
Duration of treatment for recovered participants, months	n/a	n/a	21.8 (11.7)	n/a		

Abbreviations: AN^first^, first‐episode anorexia nervosa; AN^rec^, recovered from anorexia nervosa; BMI, body mass index; CG^younger^, younger control group; CG^older^, older control group; EDE, Eating Disorder Examination; EDI, Eating Disorder Inventory‐3; RIAS, Reynolds Intellectual Assessment Scales; SD, standard deviation.

^a^ Participants older than 20 given BMI‐percentile of 20 years.

^b^ EDE data available from time of treatment for recovered AN participants, *N* = 11 (48%).

^c^ EDI data available from 31 first‐episode AN participants.

We used the Eating Disorder Risk Composite (EDRC) from the questionnaire Eating Disorder Inventory, third edition (EDI) (Garner, 2004), to examine ED symptoms in all groups. AN^first^ participants had a significantly higher score on the EDRC compared to CG^younger^ participants (*t*(48.9) = 5.25, *p* < 0.001), whereas the AN^rec^ group did not differ from CG^older^ (*t*(44) = −0.04, *p* = 0.965). Groups did not differ as to the perfectionism scale from the EDI (AN^first^ vs. CG^younger^, *t*(58) = 0.49, *p* = 0.626; AN^rec^ versus CG^older^, *t*(44) = 1.06, *p* = 0.296) (Table 1).

We screened all participants for current and lifetime presence of a mental disorder using the semi‐structured interview Schedule for Affective Disorders and Schizophrenia for School‐Age Children, Present and Lifetime version (K‐SADS‐PL) (Kaufman et al., 2000), and determined symptoms of depression and anxiety with the Beck Youth Inventory (Beck et al., 2005). The AN^first^ group scored higher on the depression and anxiety subscales than the CG^younger^ (depression, *t*(60) = 4.91, *p* < 0.001; anxiety, *t*(60) = 3.39, *p* = 0.012), whereas AN^rec^ and CG^older^ participants did not differ (depression, *t*(44) = 0.66, *p* = 0.512; anxiety, *t*(37.28) = 0.64, *p* = 0.525) (Table 1). Six participants in the AN^first^ group had a diagnosis of depression at study inclusion and six participants had an anxiety diagnosis, which was the case in one and four participants, respectively, in the AN^rec^ group. Twelve participants in the AN^rec^ group had previously been diagnosed with depression and six with an anxiety disorder.

To establish a diagnosis of an eating disorder for the AN^first^ participants and recovery status in the AN^rec^ group, we used the EDE, 16^th^ edition (Cooper & Fairburn, 1987), a semi‐structured interview focusing on psychological ED symptoms and behavior. We performed the EDE with control participants if their scores on ED items on the K‐SADS exceeded clinical threshold. The control participants were excluded if their global score on the EDE exceeded one standard deviation of community norms (Fairburn, 2008). The AN^first^ and AN^rec^ groups had similar scores on the EDE at the beginning of treatment (*t*(42) = −0.19, *p* = 0.849), however their age of onset differed, with the AN^first^ participants being 15.9 years old when starting AN treatment whereas the AN^rec^ participants had been 14.8 years old (*t*(54) = 2.58, *p* = 0.013). Four of the AN^first^ and three of the AN^rec^ participants had a binge‐eating/purging type of AN (AN‐BP) whereas the remaining participants of the groups had a restricting type of AN. The ratio of participants living with both parents and parents' education did not differ across groups (Table 1).

### Experimental task

**FIGURE 1:**
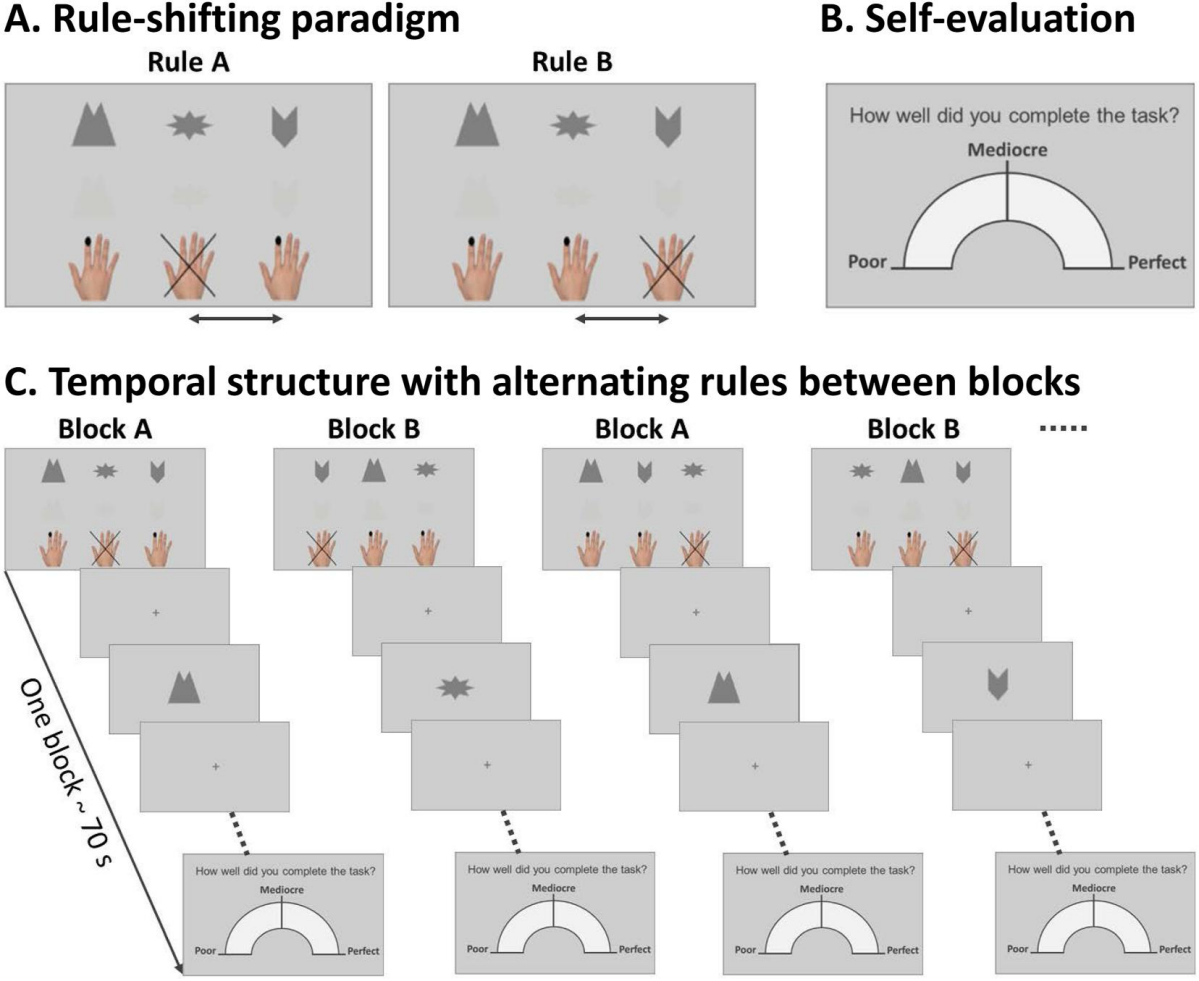
**Illustration of the Go/NoGo task.** The task included two sessions; each session consisted of 14 blocks. The participants were instructed to focus on accuracy in one session and on fast reactions in the other. The order of the sessions was counterbalanced across participants. Each session included three symbols; two of which were Go‐symbols and one was a NoGo‐symbol. This rule was learned during two practice blocks and was maintained during odd blocks. In even blocks, a Go and a NoGo‐symbol switched condition. One Go‐symbol remained a Go‐symbol throughout the session. Half of the stimuli were shown with low contrast to enhance the difficulty of the task. All 28 one‐minute blocks ended with a self‐evaluation on a continuous scale from poor to perfect.

The experimental task was a modified Go/NoGo task (Figure 1), including two sessions with 14 blocks each. The task was completed during functional magnetic resonance imaging. Participants were instructed to focus on accuracy in the “accurate session” and fast reactions in the “fast session”. We counterbalanced the order of the sessions across participants. For each session, three symbols were randomly chosen from a pool of 12. Two of the symbols were Go‐symbols for which the participants were instructed to pressing a button. The third symbol was a NoGo‐symbol and the participants had to inhibit the impulse to press the button. Prior to each session, the participants completed two learning blocks comprised of 18 trials of the same paradigm. The rule of the learning blocks was maintained for all odd blocks during the session. For the even blocks, two of the symbols switched condition; a Go‐symbol became a NoGo‐symbol and the NoGo‐symbol became a Go‐symbol. Thus, participants had to inhibit a previously learned response and change their behavior accordingly. Each symbol appeared six times during a block with an interstimulus interval of 2.5 ± 0.2 s (duration of one block ∼70 s). Half of the symbols were presented in low contrast, which was randomized within symbol type. We modified the task as described to enhance difficulty and to trigger errors. After each of the 14 blocks per session, the participants evaluated their performance on a continuous scale from poor to perfect with a total of 28 self‐evaluations per participant.

### Statistical analysis

We used SAS version 9.4 (SAS Institute Inc., Cary, NC) and R version 3.3.3 (2017‐03‐06) (R Core Team, 2017) for statistical analysis. In the two analyses of our hypotheses, we used Bonferroni correction to control for type I error (*p* < 0.025). The four groups were analyzed combined, and pairwise comparisons between the AN^first^ and CG^younger^ groups and the AN^rec^ and CG^older^ groups were analyzed post hoc.

Performance on the Go/NoGo task was used to test our first hypothesis regarding longer RTs and fewer errors in the clinical groups compared to controls. We analyzed RT from correct Go‐trials and error rate from NoGo‐trials across both sessions. In a multivariate analysis of covariance (MANCOVA), we explored differences on task performance with RT and error rate as dependent variables, group membership as an independent variable, and age as covariate.

Post hoc, we explored the association between RT and error rate using an ANCOVA. Reaction time as the dependent variable explained more of the variance in our data than error rate. Thus, we modeled RT as the dependent variable with group as the independent variable, age as covariate, and the interaction between group and error rate.

For our second hypothesis, we expected the AN^first^ and AN^rec^ groups to self‐evaluate more negatively than controls. We tested the second hypothesis by comparing the relation between task performance and self‐evaluation between groups. The self‐evaluation was reflected in a score between 0 and 1, which was transformed into *z* scores based on the CG^younger^ for the comparisons with the AN^first^ group and based on the CG^older^ in comparisons with the AN^rec^ group. We calculated a composite task performance score as the mean of the *z* scores of RT and error rate (Salthouse & Hedden, 2002). A high composite task performance score would reflect poor performance; hence we used the opposite value of the composite task performance score to subtract from the self‐evaluation *z* score. A positive self‐evaluation composite score would reflect a more positive self‐evaluation in relation to performance, while a negative score reflects a negative self‐evaluation compared to performance. We performed an ANCOVA to calculate group differences on the self‐evaluation composite score with age as covariate.

In exploratory analyses, we performed the same analyses as described above for each session separately as session instructions may have affected performance differently between groups. Further, analyses were repeated with BMI‐percentile as a covariate since low weight in itself and not AN per se may affect results. Since individuals with AN‐BP may be more impulsive than individuals with restricting AN (AN‐R), we repeated the analyses while excluding AN‐BP participants. The scales on depression and anxiety from the Beck Youth Inventory were included as covariates since the level of symptoms on these scales differed between groups.

For the Go‐trials, outliers were defined as RTs smaller than 200 ms, which are likely too fast to have been consciously processed (Amano, 2006; Woods et al., 2015). For all trials, outliers were defined as RTs larger than 1500 ms, which are likely indicative of attentional distraction. The first trial of a block was excluded for Go‐trials because a consistently longer RT suggested that participants had to reorient themselves to the task after a short break.

We excluded blocks with an error rate higher than two‐thirds in the NoGo‐trials or one‐third in the Go‐trials (total exclusions 206/3024 blocks). This ensured that the participant had understood the current rule of the task. Using paired samples *t* tests, we examined RT and error rate between sessions for each group separately.

## RESULTS

### Go/NoGo task performance

**FIGURE 2:**
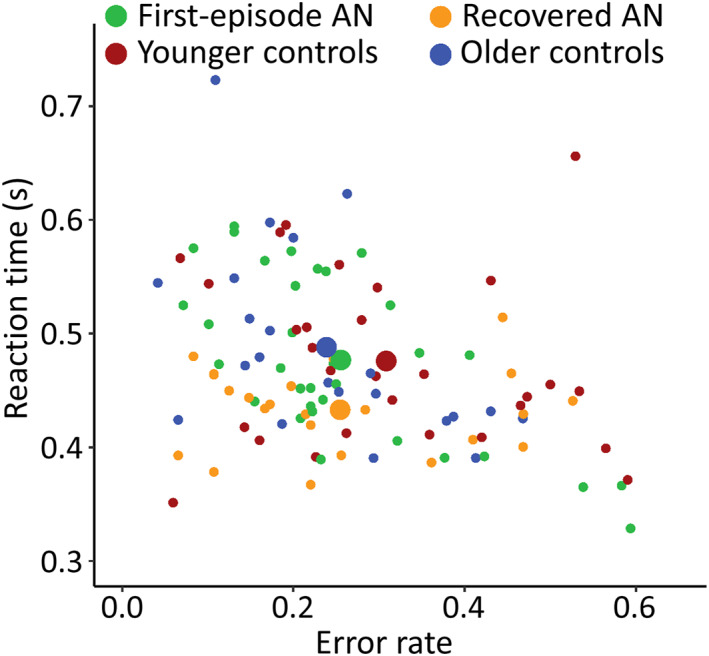
**Multivariate analysis of reaction time in seconds (y) and error rate (x).** The larger circles show the averages of the groups and small circles are the individual participants.

We explored whether any of the groups differed in any of their performance measures. A multivariate *F*‐test, with RT and error rate as dependent variables, showed that some groups performed differently on the task (*F*(3, 103) = 2.54, *p* = 0.021) (Figure 2). Repeating the multivariate *F*‐test with the AN^first^ and CG^younger^ groups alone, we did not detect any difference in performance between the groups (*F*(1, 59) = 1.35, *p* = 0.268). Further, we found that the AN^rec^ group performed better than the CG^older^ (*F*(1, 43) = 4.17, *p* = 0.022). Reaction time and error rates were comparable across the AN^first^ and CG^younger^ groups (RT, *F*(1, 59) < 0.01, *p* = 0.961; error rate, *F*(1, 59) = 2.33, *p* = 0.132) and AN^rec^ participants were significantly faster than CG^older^ (*F*(1, 43) = 8.31, *p* = 0.006) with similar error rates (*F*(1, 43) = 0.22, *p* = 0.645) (Table 2).

**TABLE 2:** Go/NoGo task performance.

	AN^first^	CG^younger^	AN^rec^	CG^older^	Test statistics	Pairwise comparisons
	Mean (SD)	Mean (SD)	Mean (SD)	Mean (SD)		
Reaction time, go‐trials (s)	0.48 (0.07)	0.48 (0.08)	0.43 (0.04)	0.49 (0.08)	MANCOVA *F*(3, 103) = 2.80, *p* = 0.044	AN^first^ versus CG^younger^, *F*(1, 59) = 0.00, *p* = 0.961
						AN^rec^ versus CG^older^, *F*(1, 43) = 8.31, *p* = 0.006
Error rate, NoGo‐trials	0.26 (0.13)	0.31 (0.15)	0.25 (0.14)	0.24 (0.12)	MANCOVA *F*(3, 103) = 1.55, *p* = 0.206	AN^first^ versus CG^younger^, *F*(1, 59) = 2.33, *p* = 0.132
						AN^rec^ versus CG^older^, *F*(1, 43) = 0.22, *p* = 0.645
Self‐evaluation raw score	0.53 (0.20)	0.66 (0.16)	0.57 (0.19)	0.65 (0.17)	ANCOVA *F*(3, 107) = 4.16, *p =* 0.008	AN^first^ versus CG^younger^, *F*(1, 61) = 7.78, *p* = 0.007
						AN^rec^ versus CG^older^, *F*(1, 45) = 2.00, *p* = 0.16
Self‐evaluation composite score	−0.97 (1.22)	7.11E‐8 (0.93)	−0.72 (0.96)	−2.69E‐7 (1.00)	ANCOVA *F*(3, 107) = 6.15, *p* < 0.001	AN^first^ versus CG^younger^, *F*(1, 61) = 11.82, *p* = 0.001
						AN^rec^ versus CG^older^, *F*(1, 45) = 5.96, *p* = 0.019

Abbreviations: AN^first^, first‐episode anorexia nervosa; AN^rec^, recovered from anorexia nervosa; CG^younger^, younger control group; CG^older^, older control group; s, seconds; SD, standard deviation.

**FIGURE 3:**
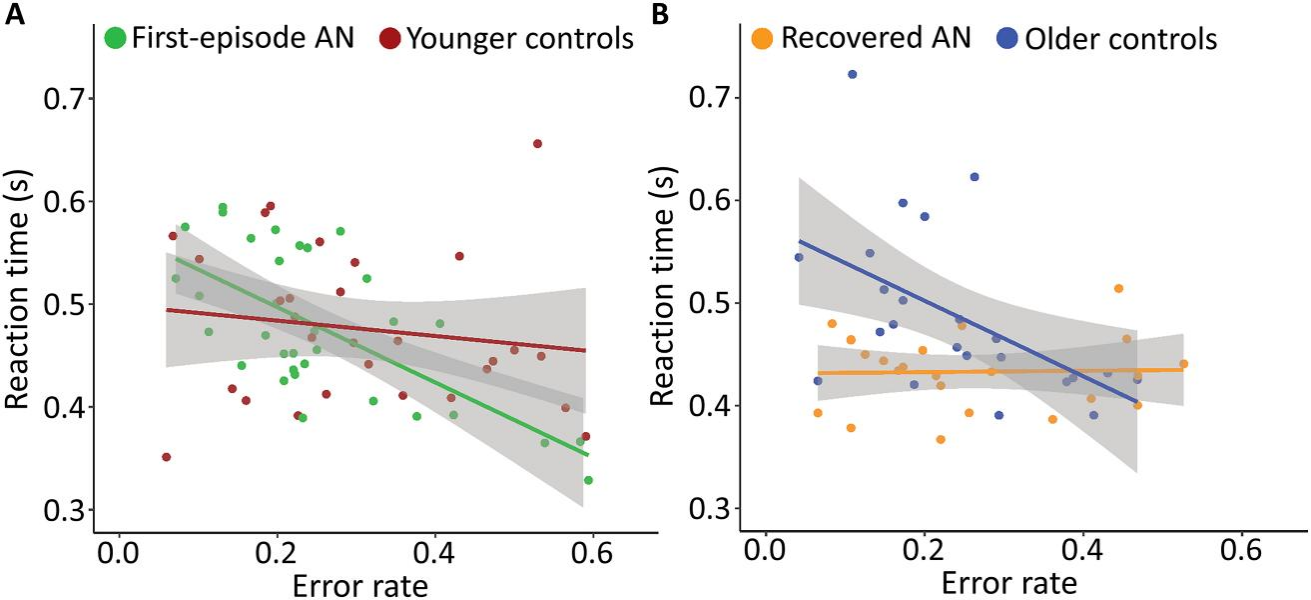
**Reaction time in seconds (y) described by error rate (x).** The lines represent the best linear fit and the shaded areas are the confidence intervals.

The association between RT and error rate was significantly more negative in the AN^first^ group compared to the CG^younger^ group (*F*(2, 61) = 9.33, *p* < 0.001) (Figure 3A). The opposite was the case for the older groups where the CG^older^ group had a significantly more negative association between RT and error rate than the AN^rec^ (*F*(2, 45) = 7.19, *p* = 0.002) (Figure 3B).

For the exploratory analyses, the pairwise comparisons between groups did not significantly change when the analyses were carried out for each session separately and when they were repeated without the AN‐BP subgroups (See Table S1). The results from the MANCOVA including all four groups revealed a slight increase in *p* values for the accurate session and when the AN‐BP subgroups were excluded (accurate session, *p* = 0.080; AN‐BP subgroups excluded, *p* = 0.058). All other findings remained significant in the exploratory analyses. BMI‐percentile was not significantly related to any of the behavioral measures (See Table S1). Age had a significant effect on error rate (*F*(1, 103) = 18.18, *p* < 0.001), but we found no group by age interaction (*F*(3, 103) = 0.48, *p* = 0.694). Anxiety and depression had significant effects on error rate (anxiety, *p* = 0.005; depression, *p* = 0.003) and not RT (anxiety, *p* = 0.075; depression, *p* = 0.068) as modeled in the MANCOVA with all four groups. The main results from the MANCOVA did not change significantly with the added covariates (See Table S1). The pairwise comparisons showed that neither depression nor anxiety affected RT or error rate in the AN^rec^ and CG^older^ groups (RT: depression, *p* = 0.466; anxiety, *p* = 0.424; error rate: depression, *p* = 0.233; anxiety, *p* = 0.570). The added covariates did not affect RT in the AN^first^ and CG^younger^ groups (depression, *p* = 0.670; anxiety, *p* = 0.720). Depression and anxiety significantly impacted the error rate in the AN^first^ and CG^younger^ groups (depression, *p* = 0.031; anxiety, *p* = 0.016) and revealed a group difference with the AN^first^ participants committing fewer errors than controls (group: *p* = 0.008 (depression), *p* = 0.009 (anxiety)). We found no group by depression or group by anxiety interactions in any of the analyses performed.

All groups displayed faster RTs in the session focusing on fast reactions and fewer errors in the session focusing on accuracy (Table 3).

**TABLE 3:** Session differences.

	Accurate session	Fast session	Paired samples *t* test
	Mean (SD)	Mean (SD)	
Reaction time, go‐trials (s)			
First‐episode AN	0.50 (0.10)	0.45 (0.06)	*t*(32) = 4.92, *p* < 0.001
Younger control group	0.51 (0.10)	0.44 (0.06)	*t*(28) = 5.67, *p* < 0.001
Recovered AN	0.45 (0.05)	0.42 (0.04)	*t*(22) = 4.16, *p* = 0.001
Older control group	0.52 (0.11)	0.46 (0.07)	*t*(22) = 3.43, *p* = 0.002
Error rate, NoGo‐trials			
First‐episode AN	0.20 (0.15)	0.31 (0.14)	*t*(32) = −5.75, *p* < 0.001
Younger control group	0.25 (0.18)	0.37 (0.15)	*t*(28) = −6.01, *p* < 0.001
Recovered AN	0.23 (0.16)	0.29 (0.14)	*t*(22) = −4.08, *p* < 0.001
Older control group	0.20 (0.13)	0.28 (0.12)	*t*(22) = −5.73, *p* < 0.001
Self‐evaluation raw score			
First‐episode AN	0.57 (0.21)	0.48 (0.20)	*t*(32) = 4.63, *p* < 0.001
Younger control group	0.70 (0.19)	0.61 (0.16)	*t*(28) = 3.54, *p* = 0.001
Recovered AN	0.60 (0.20)	0.54 (0.19)	*t*(22) = 3.21, *p* = 0.004
Older control group	0.69 (0.19)	0.61 (0.18)	*t*(22) = 2.74, *p* = 0.012
Self‐evaluation composite score			
First‐episode AN	−0.69 (1.32)	−1.25 (1.22)	*t*(32) = −4.33, *p* < 0.001
Younger control group	0.30 (1.05)	−0.30 (0.93)	*t*(28) = −4.85, *p* < 0.001
Recovered AN	−0.57 (1.03)	−0.88 (0.97)	*t*(22) = −2.64, *p* = 0.015
Older control group	0.26 (1.24)	−0.26 (1.01)	*t*(22) = −2.35, *p* = 0.028

Abbreviations: AN^first^, first‐episode anorexia nervosa; AN^rec^, recovered from anorexia nervosa; CG^younger^, younger control group; CG^older^, older control group; s, seconds; SD, standard deviation.

### Self‐evaluation

**FIGURE 4:**
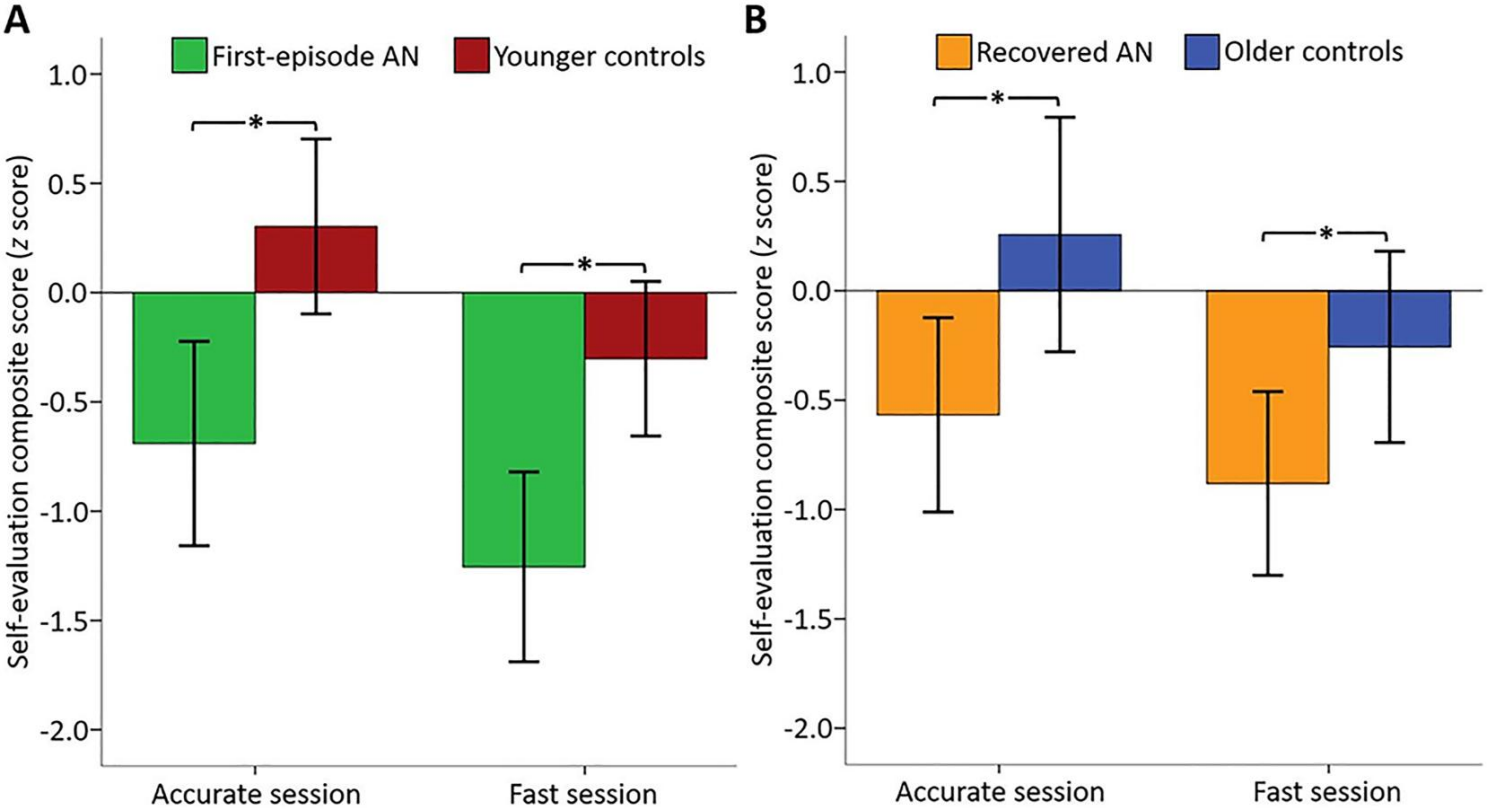
**Self‐evaluation in relation to performance.** Values represent the self‐evaluation expressed as a *z* score minus a composite task performance *z* score. The behavioral composite score is the mean of the reaction time (RT) *z* score and commission error *z* score. The opposite value of the behavioral composite score is subtracted from the self‐evaluation *z* score. Hence, a positive value reflects a positive self‐evaluation in relation to performance while a negative value represents the opposite. The error bars represent the 95% confidence interval. * significantly different at *p* < 0.05. (A,) Mean *z* score values for the accurate and fast sessions, respectively, in the first‐episode anorexia nervosa (AN) and younger control groups. (B,) Mean *z* score values for the accurate and fast sessions, respectively, in the recovered and older control groups.

We tested whether the groups differed on their self‐evaluations related to task performance. We found a main effect of group within an ANCOVA with the self‐evaluation composite score as dependent variable (self‐evaluation related to performance) (*F*(3, 107) = 6.15, *p* < 0.001). Post hoc analyses showed that participants in the AN^first^ group and the AN^rec^ group evaluated their performances significantly more negatively compared to CG^younger^ and CG^older^ confirming our second hypothesis (AN^first^ vs. CG^younger^, *F*(1, 61) = 11.82, *p* = 0.001; AN^rec^ versus CG^older^, *F*(1, 45) = 5.96, *p* = 0.019) (Table 2). Exploratory analyses showed, that the negative self‐evaluation was present in both sessions for both AN^first^ and AN^rec^ participants (AN^first^ vs. CG^younger^, accurate session, *F*(1, 61) = 10.19, *p* = 0.002; fast session, *F*(1, 61) = 11.25, *p* = 0.001; AN^rec^ versus CG^older^, accurate session, *F*(1, 45) = 5.72, *p* = 0.021; fast session, *F*(1, 45) = 4.29, *p* = 0.044) (Figure 4). Excluding the AN‐BP subgroups did not significantly alter our findings. BMI‐percentile was not significantly related to self‐evaluation. Anxiety and depression affected self‐evaluation negatively in the pairwise comparisons (AN^first^ vs. CG^younger^, depression, *p* = 0.000; anxiety, *p* = 0.005; AN^rec^ vs. CG^older^, depression, *p* = 0.004; anxiety, *p* = 0.062) and the effect was similar across groups (See Table S1).

## DISCUSSION

This study examined RT and error rate during a cognitively demanding task in adolescents with a recent onset of first‐episode AN and recovered young females, as well as the participants' self‐evaluations. We found that RT and error rate were comparable between the first‐episode AN and age‐matched controls. However, in the recovered AN group, overall task performance was better than age‐matched controls due to faster RTs. Examining our second hypothesis, we confirmed that both first‐episode and recovered AN participants evaluated their performance significantly more negatively than their respective controls.

Participants in the first‐episode AN and younger control groups performed similarly on the Go/NoGo task on RT and errors of commission but the association between the two performance measures differed indicating that the first‐episode AN group had faster RTs than the controls when the error rate was high. Similarly, previous studies on adolescents with AN did not report differences in RT and error rate when the stimuli were not related to ED‐symptomatology, however these studies did not report the association between the two performance measures (Kullmann et al., 2014; Rosval et al., 2006). Other studies have reported longer RTs and fewer errors in adults with AN and when the stimuli were related to food, weight, or shape (Bartholdy et al., 2017; Kullmann et al., 2014; Meule et al., 2011; Pieters et al., 2007). Hence, adolescents with a recent onset of AN may be able to maintain fast reactions while keeping the error rate low as long as the stimuli are neutral. A meta‐analysis including adult AN studies reported that differing RT findings may depend on task difficulty as ED‐populations tended to slow down as task difficulty increased compared to controls (Ferraro et al., 2018). The task in our study was cognitively demanding by virtue of the continuous rule switches. The meta‐analysis only included adult populations and thus it remains possible that adolescent samples with a shorter history of AN may not need to compensate to the same degree as adults. The tradeoff between RT and accuracy depends on the participants' subjective prioritization of RT or accuracy (Bartholdy et al., 2016). We instructed the participants to focus on either fast reactions or high accuracy, which may have reduced a possible difference in priorities between the participants in the first‐episode AN group and younger control group.

Participants recovered from AN performed better on the task than older control participants. They showed faster RTs but similar error rates. Moreover, the association between RT and error rate differed between the groups. The recovered AN group maintained a low error rate with fast responses while the older control group slowed down to maintain the same level of errors. These findings differ from a previous study that did not find differences in performance on a stop‐signal task between recovered and control participants (Oberndorfer et al., 2011). The recovered participants in our study were young and have recovered during adolescence or early adulthood. It is possible that they did not develop impaired response inhibition while suffering from AN or that the excessive inhibition recovers when AN symptoms improve early in life. The individuals who recover may be a selection of all patients who have better cognitive resources than individuals who do not recover fully. Future studies may look into cognitive resources as a predictor of treatment outcome and further examine the group of individuals who recover from AN at a young age.

To our knowledge, this is the first study combining a cognitive task with continuous self‐evaluation in AN, thus adding to the sparse body of literature on behavioral perfectionism. First‐episode AN participants evaluated their performance more negatively than matched control participants. This finding complements the behavioral evidence to the large body of self‐report studies (Boone et al., 2014; Forbush et al., 2007; Levinson et al., 2013) and shows that perfectionism also applies to the adolescent's performance in settings unrelated to ED‐behavior.

Similarly, the recovered participants evaluated their performance more negatively than control participants. Previous studies have found that perfectionism is one of the only studied factors that remains high during the process of recovery (Nilsson et al., 2008; Schneider et al., 2009; Srinivasagam et al., 1995) but findings are inconsistent. Contrary to previous studies, our study included a young group of recovered females using strict criteria for recovery and moreover, we measured perfectionism behaviorally and not exclusively with a questionnaire. The EDI perfectionism scale did not reveal any significant differences between groups in this study. The six‐item scale was constructed as a unidimensional scale but later factor analyses have revealed two scales, self‐oriented and socially prescribed perfectionism (Garner, 2004; Lampard et al., 2012; Sherry et al., 2003). No norms are available for the subscales and possibly the subscales could reveal group differences. Especially self‐oriented perfectionism has been found to be related to dietary restraint as well as weight and shape concern in AN as opposed to the dimension on socially prescribed perfectionism (Bardone‐Cone, 2007; Lampard et al., 2012).

The differing findings on self‐evaluation and the EDI perfectionism scale may reflect different markers of the complex construct of perfectionism. The negative self‐evaluations may reflect a focus on failure when not meeting their own standards (Egan et al., 2011; Shafran et al., 2002). This aspect is only one part out of several on the EDI perfectionism scale and is possibly part of the explanation why our findings differ.

Our findings on negative self‐evaluation could also represent an aspect of negative self‐concept including negative self‐esteem. Self‐concept can be characterized as a personality trait and, hence, more stable over time (Button & Warren, 2002; Critchfield & Benjamin, 2010), which is in line with our finding where both clinical groups self‐evaluate their actions more negatively than control participants. Other studies have found that self‐esteem and a negative self‐concept can be improved during ED treatment and are correlated to recovery from ED (Bardone‐Cone, Schaefer, et al., 2010; Gezelius et al., 2016; Kelly & Tasca, 2016; Petersson et al., 2021). Self‐esteem may thus play a central role in the relationship between perfectionism and several ED‐symptoms in adulthood, however with scarce evidence (Puttevils et al., 2019). In our study, irrespectively of the young age of the recovered participants and the strict criteria in defining recovery, the participants self‐evaluated significantly more negatively than control participants. This underscores the importance of supplementing self‐report with behavioral measures to individualize treatment. We cannot exclude that these traits may play an important role for the risk of relapse and they thus warrant a more thorough examination.

A previous study has found that perfectionism levels were higher when individuals with an ED suffered from comorbid obsessive‐compulsive disorder (Halmi et al., 2005) and perfectionism has been described as a risk factor in anxiety and depression (Egan et al., 2011; Handley et al., 2015; Leitenberg et al., 1986; Lloyd et al., 2015; Sassaroli et al., 2008). We found that the level of anxiety and depression affected performance, mainly the error rate in the currently ill AN participants, and similarly to previous findings, we showed that anxiety and depression impacted self‐evaluation negatively (Shafran et al., 2002). The inclusion of comorbid symptoms did not affect our main analyses, and anxiety and depression impacted all groups in a similar fashion. Thus, AN may be primarily driving our results but the effect of comorbid symptoms on behavior emphasizes the complexity of the disorder.

Inhibition and impulsivity have been described on a diagnostic continuum from AN‐R on one end to binge‐eating on the other (Wierenga et al., 2014; Wu et al., 2013). In our study, the results were not altered when excluding participants with AN‐BP from the first‐episode and recovered AN groups. Not all studies reported behavioral differences on inhibition tasks between the two AN subtypes even though individuals with AN‐BP tend to self‐report higher impulsivity than AN‐R (Claes et al., 2006, 2012; Farstad et al., 2016; Galimberti et al., 2012; Lock et al., 2011). We did not aim to analyze subgroup differences and cannot rule out that the AN‐BP subgroups performed differently from participants with AN‐R. If differences were present, the AN‐BP subgroups were too small to impact our results significantly.

A previous study has reported a positive correlation between RT and BMI in adults with current AN (Claes et al., 2012). In our study, BMI‐percentile did not correlate significantly to RT, error rate, nor self‐evaluation in any group. Even though self‐evaluation is based upon eating, shape and/or weight for many individuals with AN, self‐evaluation in areas not related to ED‐symptomatology seems to be negatively influenced even when individuals have recovered from AN. Several studies have shown that high levels of perfectionism and a negative self‐concept measured during the treatment phase predicted a poorer prognosis and lowered the individual's chances of recovery (Bardone‐Cone, Schaefer, et al., 2010; Bizeul et al., 2001; Rigaud et al., 2011). Few studies have examined relapse from remission in adolescent treatment studies and the findings point to low rates of relapse as long as full recovery has been achieved (Eisler et al., 2007; Le Grange et al., 2014). This indicates that our sample of recovered young females may have good chances of staying recovered and the possible implications of the negative self‐evaluation measured in this study need further investigation.

The majority of studies of perfectionism in AN during treatment have been conducted with adult samples using cognitive behavioral therapy enhanced with a perfectionism or self‐esteem module (CBT‐E), however, findings are inconsistent (Bardone‐Cone, Sturm, et al., 2010; Byrne et al., 2011; Farstad et al., 2016; Goldstein et al., 2014; Handley et al., 2015; Lloyd et al., 2015). One study concluded that CBT‐E did not lower perfectionism scores more than regular CBT (Goldstein et al., 2014) and another study found that concern over mistakes did not improve (Levinson et al., 2017). Other studies showed that an add‐on treatment focusing on perfectionism or self‐esteem lowered the corresponding self‐report scores, however, these studies have not included active control groups (Berthod et al., 2014; Byrne et al., 2011; Handley et al., 2015; Lloyd et al., 2015; Tchanturia et al., 2016). Family‐based treatment is the recommended treatment of choice for adolescents who present for treatment for the first time (National Institute for Health and Care Excellence, 2017). Family‐based treatment is usually divided into three phases whereof the third phase covers other aspects of the adolescent's life than AN‐related topics (Lock & Le Grange, 2005). One of few treatment studies of perfectionism during adolescence added CBT‐P to phase two of Family‐based treatment and found a reduction in perfectionism following the module (Hurst & Zimmer‐Gembeck, 2018). The study did not compare the effect to treatment as usual, which limits possible conclusions. Together, these findings emphasize the need for well‐controlled treatment studies focusing on perfectionism and self‐esteem and their impact on outcome.

### Strengths and limitations

Among the major strengths of this study are the four groups of participants. The participants with first‐episode AN had a short illness duration, which avoided the possible confounds of chronicity. We used strict criteria in defining recovery, and the control participants were closely matched to age in both clinical groups. All participants underwent a thorough clinical assessment.

Although the design of a cross‐sectional study limits interferences concerning developmental aspects and the course of disorder, the examination of recovered individuals allows exploring state versus trait characteristics to a certain extent.

Furthermore, our recovered group may represent a subgroup of the adolescents that present for treatment for AN. These individuals had a good outcome and did not suffer from other psychiatric disorders that impacted daily function significantly. In this study, symptom severity at the beginning of treatment was similar across the first‐episode AN and recovered AN group but age of AN onset was lower in the recovered group than in the first‐episode AN group. Younger age at onset has been shown as a predictor of better outcome in some studies (Berkman et al., 2007; Eisler et al., 1997).

We included individuals with an AN‐BP subtype in the clinical groups to avoid a too narrow diagnostic scope and showed in our analyses that the diagnostic heterogeneity did not unduly influence the results.

The development of a novel way to examine perfectionism adds to the field of how we may examine perfectionism and self‐evaluation in future studies. Our study is limited by the fact that a continuous pursuit of unrealistically high goals is difficult to measure behaviorally in a single session. We added self‐evaluations to be completed continuously throughout the task, but the self‐evaluations are limited to the experimental setting. This emphasizes the need for the combination of methods to understand the construct of perfectionism more fully.

## AUTHOR CONTRIBUTIONS

**Tine Schuppli Hjerresen**: Data curation; Formal analysis; Investigation; Methodology; Project administration; Visualization; Writing – original draft. **Mette Bentz**: Data curation; Investigation; Methodology; Project administration; Validation; Writing – review & editing. **Ayna Baladi Nejad**: Formal analysis; Methodology; Writing – review & editing. **Estelle Raffin**: Conceptualization; Formal analysis; Investigation; Methodology; Validation; Writing – review & editing. **Kasper Winther Andersen**: Formal analysis; Methodology; Validation; Visualization; Writing – review & editing. **Oliver James Hulme**: Conceptualization; Methodology; Validation; Writing – review & editing. **Hartwig Roman Siebner**: Conceptualization; Formal analysis; Funding acquisition; Investigation; Methodology; Resources; Supervision; Validation; Writing – review & editing. **Kerstin Jessica Plessen**: Conceptualization; Formal analysis; Funding acquisition; Investigation; Methodology; Resources; Supervision; Validation; Visualization; Writing – review & editing.

## CONFLICT OF INTEREST STATEMENT

Hartwig Roman Siebner was supported by a 5‐year professorship in precision medicine at the Faculty of Health Sciences and Medicine, University of Copenhagen, which is sponsored by the Lundbeck Foundation (Grant Nr. R186‐2015‐2138). He has received honoraria as speaker from Sanofi Genzyme, Denmark, Lundbeck AS, Denmark, and Novartis, Denmark, as consultant from Sanofi Genzyme, Denmark, and Lundbeck AS, Denmark, and as editor‐in‐chief (Neuroimage Clinical) and senior editor (NeuroImage) from Elsevier Publishers, Amsterdam, The Netherlands. He has received royalties as book editor from Springer Publishers, Stuttgart, Germany and from Gyldendal Publishers, Copenhagen, Denmark. Oliver James Hulme was funded by a Novo Nordisk Foundation Exploratory Interdisciplinary Synergy Grant, ref NNF20OC0064869. All other authors report no competing or potential conflicts of interest.

## Ethical considerations

The authors obtained study approval from the regional Scientific Ethical Committees (project number H‐2‐2012‐027) and The Danish Data Protection Agency and informed consent from participants and legal caretakers according to the guidelines of the Danish Health and Medicines Authority.

## Supporting information

Supplementary Material

## Data Availability

Data is available in anonymized form from the corresponding author on reasonable request.
