# Supplementary material for: Performing well but not appreciating it – A trait feature of anorexia nervosa
Source: JCPP Adv. 2023 Sep 20;4(1):e12194. doi: 10.1002/jcv2.12194 (PMC10933629; doi:10.1002/jcv2.12194)
Supplement: Supplementary file 1 — Supplementary Material [file JCV2-4-e12194-s001.docx]

# Supporting Information

**Table S1. Exploratory analyses.** Main analyses for each session separately, without the participants with AN-BP, and BMI-percentile, anxiety, and depression as covariates.

|  | AN^first^ mean (SD) | CG^younger^ mean (SD) | AN^rec^ mean (SD) | CG^older^ mean (SD) | Test statistics | Pairwise comparisons |
| --- | --- | --- | --- | --- | --- | --- |
| Accurate session |  |  |  |  |  |  |
| Overall task performance |  |  |  |  | MANCOVA *F*(3, 103) = 1.91, *p* = .080 | AN^first^ vs. CG^younger^, *F*(1, 59) = 1.05, *p* = .355  AN^rec^ vs. CG^older^, *F*(1, 43) = 3.71, *p* = .033 |
| Reaction time, Go-trials (s) | 0.50 (0.10) | 0.51 (0.10) | 0.45 (0.05) | 0.52 (0.11) | MANCOVA *F*(3, 103) = 2.71, *p* = .048 | AN^first^ vs. CG^younger^, *F*(1, 59) = 0.08, *p* = .782  AN^rec^ vs. CG^older^, *F*(1, 43) = 7.60, *p* = .009 |
| Error rate, NoGo-trials | 0.20 (0.15) | 0.25 (0.18) | 0.23 (0.16) | 0.20 (0.13) | MANCOVA *F*(3, 103) = 0.81, *p* = .493 | AN^first^ vs. CG^younger^, *F*(1, 59) = 1.36, *p* = .248  AN^rec^ vs. CG^older^, *F*(1, 43) = 0.67, *p* = .418 |
| Self-evaluation composite score | -0.69 (1.32) | 0.30 (1.05) | -0.57 (1.03) | 0.27 (1.23) | ANCOVA *F*(3, 107) = 5.61, *p* = .001 | AN^first^ vs. CG^younger^, *F*(1, 61) = 10.19, *p* = .002  AN^rec^ vs. CG^older^, *F*(1, 45) = 5.72, *p* = .021 |
| Fast session |  |  |  |  |  |  |
| Overall task performance |  |  |  |  | MANCOVA *F*(3, 103) = 2.55, *p* = .021 | AN^first^ vs. CG^younger^, *F*(1, 59) = 1.49, *p* = .234  AN^rec^ vs. CG^older^, *F*(1, 43) = 2.84, *p* = .070 |
| Reaction time, Go-trials (s) | 0.45 (0.06) | 0.44 (0.06) | 0.42 (0.04) | 0.46 (0.07) | MANCOVA *F*(3, 103) = 1.92, *p* = .131 | AN^first^ vs. CG^younger^, *F*(1, 59) = 0.27, *p* = .603  AN^rec^ vs. CG^older^, *F*(1, 43) = 5.34, *p* = .026 |
| Error rate, NoGo-trials | 0.31 (0.14) | 0.37 (0.15) | 0.29 (0.14) | 0.28 (0.12) | MANCOVA *F*(3, 103) = 2.67, *p* = .052 | AN^first^ vs. CG^younger^, *F*(1, 59) = 3.03, *p* = .087  AN^rec^ vs. CG^older^, *F*(1, 43) = 0.01, *p* = .921 |
| Self-evaluation composite score | -1.25 (1.22) | -0.30 (0.93) | -0.88 (0.97) | -0.26 (1.01) | ANCOVA *F*(3, 107) = 5.39, *p =* .002 | AN^first^ vs. CG^younger^, *F*(1, 61) = 11.25, *p* = .001  AN^rec^ vs. CG^older^, *F*(1, 45) = 4.29, *p* = .044 |
| AN-BP  excluded |  |  |  |  |  |  |
| Overall task performance |  |  |  |  | MANCOVA *F*(3, 96) = 2.07, *p* = .058 | AN^first^ vs. CG^younger^, *F*(1, 55) = 1.18, *p* = .334  AN^rec^ vs. CG^older^, *F*(1, 40) = 3.48, *p* = .041 |
| Reaction time, Go-trials (s) | 0.47 (0.07) | 0.48 (0.08) | 0.43 (0.04) | 0.49 (0.08) | MANCOVA *F*(3, 96) = 2.37, *p* = .075 | AN^first^ vs. CG^younger^, *F*(1, 55) = 0.12, *p* = .731  AN^rec^ vs. CG^older^, *F*(1, 40) = 7.12, *p* = .011 |
| Error rate, NoGo-trials | 0.26 (0.14) | 0.31 (0.15) | 0.26 (0.15) | 0.24 (0.12) | MANCOVA *F*(3, 96) = 1.31, *p* = .276 | AN^first^ vs. CG^younger^, *F*(1, 55) = 1.48, *p* = .229  AN^rec^ vs. CG^older^, *F*(1, 40) = 0.47, *p* = .498 |
| Self-evaluation composite score | -0.99 (1.25) | 7.11E-8 (0.93) | -0.78 (1.01) | -2.69E-7 (1.00) | ANCOVA *F*(3, 100) = 5.73, *p* = .001 | AN^first^ vs. CG^younger^, *F*(1, 57) = 10.70, *p* = .002  AN^rec^ vs. CG^older^, *F*(1, 42) = 5.83, *p* = .021 |
| BMI as  covariate |  |  |  |  |  |  |
| Overall task performance |  |  |  |  | MANCOVA *F*(1, 102) = 2.06, *p* = .132 | AN^first^ vs. CG^younger^, *F*(1, 58) = 1.66, *p* = .199  AN^rec^ vs. CG^older^, *F*(1, 42) = 1.85, *p* = .170 |
| Reaction time, Go-trials (s) |  |  |  |  | MANCOVA *F*(1, 102) = 3.91, *p* = .051 | AN^first^ vs. CG^younger^, *F*(1, 58) = 1.02, *p* = .317  AN^rec^ vs. CG^older^, *F*(1, 42) = 3.36, *p* = .074 |
| Error rate, NoGo-trials |  |  |  |  | MANCOVA *F*(1, 102) = 1.41, *p* = .238 | AN^first^ vs. CG^younger^, *F*(1, 58) = 3.28, *p* = .075  AN^rec^ vs. CG^older^, *F*(1, 42) = 0.00, *p* = .980 |
| Self-evaluation composite score |  |  |  |  | ANCOVA *F*(1, 107) = 2.74, *p =* .101 | AN^first^ vs. CG^younger^, *F*(1, 61) = 0.85, *p* = .360  AN^rec^ vs. CG^older^, *F*(1, 45) = 2.05, *p* = .160 |
| Anxiety as  covariate |  |  |  |  |  |  |
| Overall task performance |  |  |  |  | MANCOVA *F*(1, 102) = 4.33, *p* = .016 | AN^first^ vs. CG^younger^, *F*(1, 58) = 4.24, *p* = .019  AN^rec^ vs. CG^older^, *F*(1, 42) = 0.69, *p* = .508 |
| Reaction time, Go-trials (s) |  |  |  |  | MANCOVA *F*(1, 102) = 0.40, *p* = .532 | AN^first^ vs. CG^younger^, *F*(1, 58) = 1.02, *p* = .317  AN^rec^ vs. CG^older^, *F*(1, 42) = 3.36, *p* = .074 |
| Error rate, NoGo-trials |  |  |  |  | MANCOVA *F*(1, 102) = 7.76, *p* = .007 | AN^first^ vs. CG^younger^, *F*(1, 58) = 7.39, *p* = .009  AN^rec^ vs. CG^older^, *F*(1, 42) = 0.32, *p* = .576 |
| Self-evaluation composite score |  |  |  |  | ANCOVA *F*(1, 107) = 3.78, *p =* .013 | AN^first^ vs. CG^younger^, *F*(1, 61) = 4.77, *p* = .033  AN^rec^ vs. CG^older^, *F*(1, 45) = 5.47, *p* = .024 |
| Depression as  covariate |  |  |  |  |  |  |
| Overall task performance |  |  |  |  | MANCOVA *F*(1, 102) = 4.93, *p* = .009 | AN^first^ vs. CG^younger^, *F*(1, 58) = 3.54, *p* = .036  AN^rec^ vs. CG^older^, *F*(1, 42) = 1.40, *p* = .259 |
| Reaction time, Go-trials (s) |  |  |  |  | MANCOVA *F*(1, 102) = 3.10, *p* = .030 | AN^first^ vs. CG^younger^, *F*(1, 58) = 1.02, *p* = .317  AN^rec^ vs. CG^older^, *F*(1, 42) = 3.36, *p* = .074 |
| Error rate, NoGo-trials |  |  |  |  | MANCOVA *F*(1, 102) = 3.79, *p* = .013 | AN^first^ vs. CG^younger^, *F*(1, 58) = 7.60, *p* = .008  AN^rec^ vs. CG^older^, *F*(1, 42) = 0.34, *p* = .565 |
| Self-evaluation composite score |  |  |  |  | ANCOVA *F*(1, 107) = 2.87, *p =* .040 | AN^first^ vs. CG^younger^, *F*(1, 61) = 1.53, *p* = .222  AN^rec^ vs. CG^older^, *F*(1, 45) = 5.64, *p* = .022 |
